# Acceptance of artificial intelligence clinical assistant decision support system to prevent and control venous thromboembolism among healthcare workers: an extend Unified Theory of Acceptance and Use of Technology Model

**DOI:** 10.3389/fmed.2025.1475577

**Published:** 2025-02-11

**Authors:** Jingxian Wang, Yun Zhou, Kai Tan, Zhigang Yu, You Li

**Affiliations:** ^1^School of Management, Shanxi Medical University, Jinzhong, China; ^2^The Fifth Clinical Medical College, Shanxi Medical University, Taiyuan, China; ^3^Shanxi Provincial Integrated Traditional Chinese Medicine and Western Medicine Hospital, Taiyuan, China; ^4^Medical Service Division, Shanxi Provincial People's Hospital, Taiyuan, China

**Keywords:** clinical decision support system, healthcare worker, behavioral intention, venous thromboembolism, the Unified Theory of Acceptance and Use of Technology

## Abstract

**Background:**

Venous thromboembolism (VTE) is an important global health problem and the third most prevalent cardiovascular disorder. It has been proven that computerized tools were helpful in the prevention and control of VTE. However, studies that focused on the acceptance of computerized tools for VTE prevention among healthcare workers were limited.

**Objective:**

This study aims to explore what factors are influencing healthcare workers’ acceptance of the Artificial Intelligence Clinical Assistant Decision Support System (AI-CDSS) for VTE prevention based on the extended Unified Theory of Acceptance and Use of Technology (UTAUT).

**Methods:**

We conducted a cross-sectional survey among healthcare workers in three grade-A tertiary hospitals in Shanxi, China. Statistically, the hypothesized model was evaluated by AMOS structural equation modeling.

**Results:**

510 (72.86%) valid surveys were collected in total. The results showed that performance expectancy (*β* = 0.45, *P* < 0.001), effort expectancy (*β* = 0.21, *P* < 0.001), and top management support (*β* = 0.30, *P* < 0.001) positively influenced healthcare workers’ intention. Top management support was an antecedent of performance expectancy (*β* = 0.41 , *P* < 0.001), social influence (*β* = 0.57, *P* < 0.001), effort expectancy (*β* = 0.61, *P* < 0.001), and information quality (*β* = 0.59, *P* < 0.001). In addition, Social influence positively influenced performance expectancy (*β* = 0.52, *P* < 0.001), and information quality positively influenced system quality (*β* = 0.65, *P* < 0.001). Social influence did not influence nurses’ behavioral intention (*β* = 0.06, *p* = 0.376), but negatively influenced clinicians’ behavioral intention in the model (*β* = −0.19, *P* < 0.001). System quality positively influenced nurses’ behavioral intention; (*β* = 0.16, *P* < 0.001), and information quality positively influenced clinicians’ behavioral intention (*β* = 0.15, *p* = 0.025).

**Conclusion:**

With this model explaining 76.3% variance of the behavioral intention variable, this study could be useful as a reference for hospital administrators to evaluate future developments and facilitate the implementation of AI-CDSS for VTE prevention.

## 1 Introduction

### 1.1 Background

Venous thromboembolism (VTE) is an important global health problem and the third most prevalent cardiovascular disorder (1, 2). VTE has a high incidence rate worldwide. In North America, Western Europe, southern Latin America (Argentina), and Australia, the yearly incidence rates of VTE vary from 0.75 to 2.69 per 1,000 people (3, 4). Compared to African-American populations, the overall VTE incidence in Asians and Asian Americans may be lower (5). However, with lifestyle changes, longer life spans, widespread use and increased sensitivity of imaging examination techniques (6), VTE incidence is increasing in Asian countries (5, 7).

In China, previous studies reported that the total yearly incidences of PE and DVT were 3.9 and 17.1 per 100,000 population, respectively, in 2001 (8). In 2011, the total yearly incidences of PE and DVT increased to 8.7 and 30.0 per 100,000 population, respectively, in China (7). Another study also found that the rates of VTE grew sharply in the south and north of China (9). In addition, Chinese patients’ hospitalization rate of VTE has gone up with time, rising from 3.2 per 100,000 people to 17.5 per 100,000 people (9). In total, VTE is becoming a common issue in China.

Venous thromboembolism is a preventable disease. Recommended preventive measures of guidelines help to lower the rates of VTE (10). Common preventive measures include pharmacological prevention such as the use of anticoagulant agents (low molecular weight heparin, etc.) and mechanical prevention such as the use of compression stockings and so on (10). Nevertheless, the prophylaxis rate nowadays is rather low (11). According to research, many hospitalized patients did not receive CHEST-recommended (12, 13) VTE prophylaxis in China, and only 14.3% of patients at risk got some prophylaxis, with about 10.3% obtaining the necessary prophylaxis. This means that physicians frequently fail to follow guidelines, thus indicating a huge gap between current Chinese clinical practice and mostly Western consensus guidelines (14).

Many interventions were introduced to help the implementation of thromboprophylaxis, including computerized tools (e.g., electronic reminders), education interventions (e.g., courses), audit and feedback strategies, paper-based tools (e.g., preprinted orders, posters), and multifaceted interventions (combination of interventions such as education, alert, and audit and feedback) (15–17). Studies showed that computerized tools intervention were useful for increasing VTE prophylaxis (17, 18). The computerized tool to prevent and control VTE in this paper refers to the Artificial Intelligence Clinical Assistant Decision Support System (AI-CDSS), which is a big data governance system based on natural language processing, machine learning, knowledge mapping, and other technologies. It is integrated into the hospital information system, and interfaces with laboratories, imaging detection and electronic medical records systems. For clinicians, it is a system which could help them to make a preliminary VTE risk assessment and intelligently provide suggestions on preventive measures based on the assessment results and the latest clinical practice guidelines and expert consensus. For nurses, it is a system which could help them automatically assess the VTE risk of patients, prompt them to implement clinicians’ instruction in time and offer them the patient personalized education materials about VTE (19).

However, when introducing a new artificial intelligence system into the hospital where did not use before, the acceptance of the system among nurses and clinicians is hindered by many factors (20, 21). It is vital to understand nurses’ and clinicians’ willingness to use the AI-CDSS and explore the influencial factors, thus helping the improvement of system design, promoting the implementation of the system, and helping the prevention of VTE.

Previous studies mainly focused on the effect of VTE intervention measures, including education interventions (22), information-based interventions (18, 23) and so on. However, few studies explored the acceptance of intervention measures among healthcare workers. In addition, there are some studies on the intention of healthcare workers to adopt the new system (24), but few studies on the acceptance of VTE prevention and control information systems among healthcare workers and even fewer studies on the acceptance of VTE information systems based on the extended the Unified Theory of Acceptance and Use of Technology (UTAUT) model among healthcare workers. Therefore, the purpose of this study is to explore the factors impacting nurses’ and clinicians’ acceptance of AI-CDSS to prevent and control VTE, and to evaluate the correlations among different factors based on the extended UTAUT model.

### 1.2 Theoretical foundation and hypothesis development

The UTAUT model, Information System Success Model (ISSM), and the top management support variable were used in this research. UTAUT can adapt to different scenarios of medical care by supplementing contextual constructs (25–27). However limited studies have attempted to add external factors such as system quality and information quality in ISSM into the original UTAUT model, and discuss the relationship among different variables. Furthermore, a study suggested that the antecedents of the main variables could better explain the acceptance of technology (28). However, there is little known about the influence of top management support as an antecedent on core variables in UTAUT. Therefore, this study adopted top management support as an antecedent in extending UTAUT to study further. Therefore, these three variables are added to extend the UTAUT model. It is the first attempt to integrate the top management support variable, ISSM, and UTAUT model.

#### 1.2.1 The Unified Theory of Acceptance and Use of Technology

UTAUT was developed by Venkatesh and Davis (29). It is considered the widely used and the most comprehensive theoretical model that explains around 70% of the behavioral intention variance of using technology and around 50% of the actual use variance (30, 31). UTAUT integrates eight classical models, involving TAM, TRA, TPB, MPC, IDT, MM, SCT, and C-TAM-TPB (29). It gradually expands into four core variables including performance expectancy, effort expectancy, social influence, and facilitating conditions, and the outcome variable: behavioral intention (29). The first three core variables and the outcome variable in UTAUT are retained in this study. We used the variable of behavioral intention to represent the acceptance of the new system.

##### 1.2.1.1 Performance expectancy

Performance expectancy refers to the degree to which someone believes that using the system will help to achieve improvements in job performance (29). Studies have found that performance expectancy positively affected individuals’ behavioral intentions (25, 32). Regarding the prevention and control of VTE, AI-CDSS helps to capture medical record information and then evaluates it instead of manual methods, which can save much time and energy, promote work efficiency, and improve the quality of work. Therefore, if medical staff perceive the usefulness of the AI-CDSS, they prefer to adopt it when working. This paper proposed Hypothesis 1:

*Hypothesis 1*: Performance expectancy positively influences healthcare workers’ behavioral intention to use AI-CDSS for VTE prevention.

##### 1.2.1.2 Effort expectancy

Effort expectance is the extent to which the system is easy to use (29). Studies showed that effort expectancy positively influenced the degree to which medical staff accept new technologies (25, 26, 32). If medical staff recognize AI-CDSS is easy to use, they would like to adopt it. Therefore, the paper proposed Hypothesis 2:

*Hypothesis 2*: Effort expectance positively influences healthcare workers’ behavioral intention to use AI-CDSS for VTE prevention.

##### 1.2.1.3 Social influence

Social influence is the degree to which people recognize that others who are important believe they ought to use the new system (29). A potential user is willing to use a new system if others around them use it. Many studies have shown that social influence influenced behavioral intention (25, 33). Therefore, the hypotheses were as follows:

*Hypothesis 3*: Social influence positively influences healthcare workers’ behavioral intention to use AI-CDSS for VTE prevention.

*Hypothesis 4*: Social influence positively influences healthcare workers’ Performance expectancy.

#### 1.2.2 The ISSM model

DeLone and McLean developed ISSM in 1992 (34), and they published the updated model in 2003 (35) after suggested extension and modification to the initial model (36). The updated model increases to six interconnected and interdependent variables, and it is generally considered a more extensively used model of information systems success (37). The model was applied to the medical area (38, 39). System quality and information system are retained in this study.

##### 1.2.2.1 System quality

System quality connects with whether or not the system has defects, or has high maintainability, consistent user interface, high quality of document, and is easy to use (36). A study showed system quality correlated with behavior intention (35), and more studies showed that system quality positively affected behavioral intentions (33, 40). Therefore, the study proposed Hypothesis 5:

*Hypothesis 5*: System quality positively influences healthcare workers’ behavioral intention to use AI-CDSS for VTE prevention.

##### 1.2.2.2 Information quality

Information quality is “concerned with such issues as the relevance, timeliness, and accuracy of the information generated by an information system” (36). Studies showed that information quality positively influenced behavioral intention (41, 42). If AI-CDSS provides healthcare workers with up-to-date, precise, and sufficient information, they intend to use the system. Therefore, the hypotheses were as follows:

*Hypothesis 6*: Information quality positively influences healthcare workers’ behavioral intention to use AI-CDSS for VTE prevention.

*Hypothesis 7*: Information quality positively influences system quality of AI-CDSS.

#### 1.2.3 Top management support

Top management support of information systems is the extent to which senior administrators realize the information system is vital and engaged in system implementation (43). It means explicit and active support is regarded as a key factor in implementing a new system successfully (44, 45). Studies showed that top management support positively influenced behavior or behavioral intention (46, 47). It is widely acknowledged that introducing a new system in an organization is complex and challenging. Top management would ensure organizational resources are available, comprehensively consider the positions and relations of numerous different people in the organization, and then take appropriate measures to persuade and inspire users to adopt the new system (48, 49). Therefore, top management support may influence users’ perceived ease and perceived usefulness to a new system. It also influences the social environment around users when they work. In addition, administrators would ensure the design of the system is suitable and consider many issues, including the advanced characteristics of the new system, and the safety and security of system data (47, 50). It means that top management will pay attention to the quality of information and systems when introducing a new system. The paper proposed the following hypotheses:

*Hypothesis 8*: Top management support positively influences healthcare workers’ behavioral intention to use AI-CDSS for VTE prevention.

*Hypothesis 9*: Top management support positively influences healthcare workers’ performance expectancy.

*Hypothesis 10*: Top management support positively influences healthcare workers’ effort expectancy.

*Hypothesis 11*: Top management support positively influences social influence in the organization.

*Hypothesis 12*: Top management support positively influences system quality of AI-CDSS.

*Hypothesis 13*: Top management support positively influences information quality of AI-CDSS.

**Figure 1:**
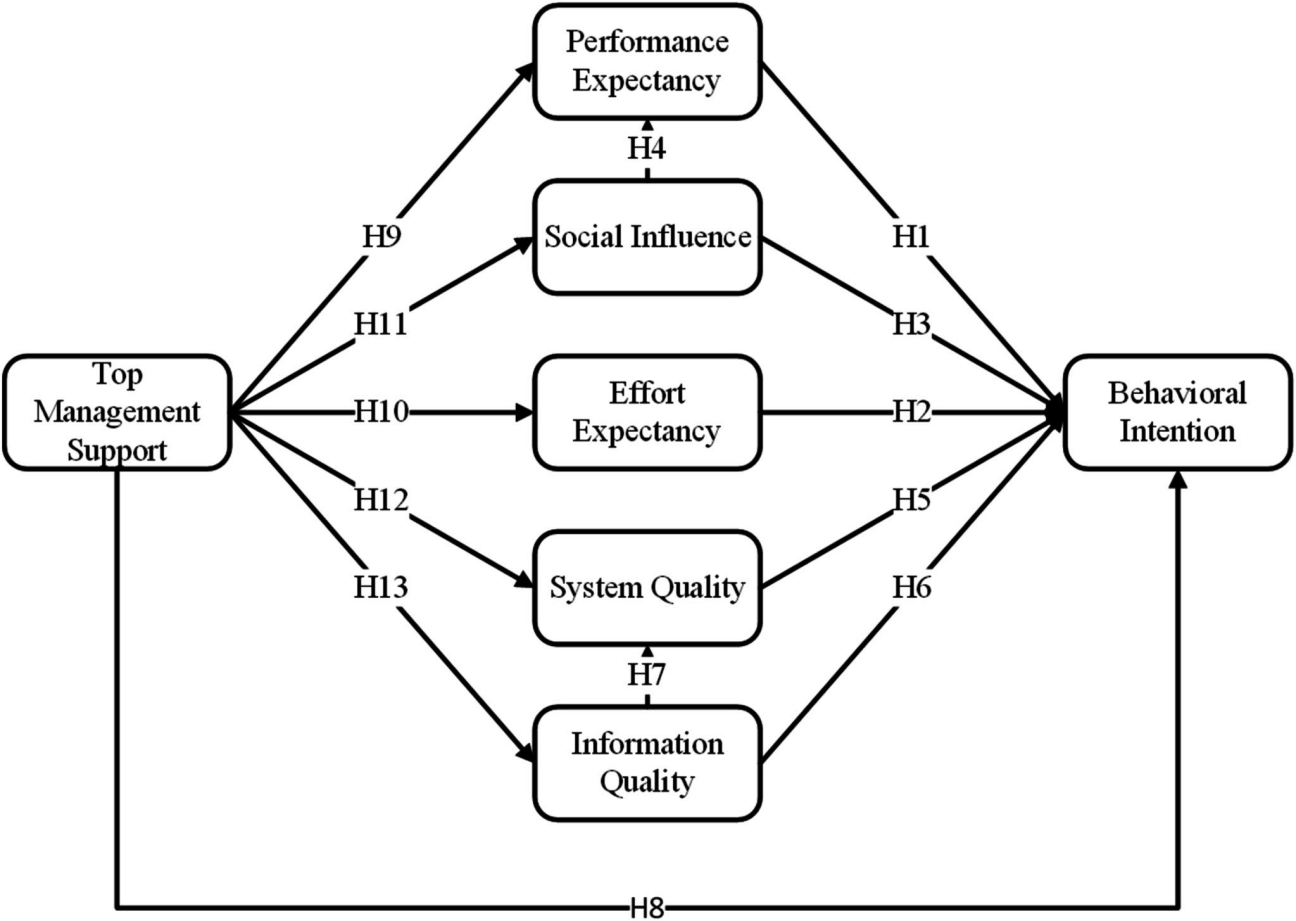
Conceptualized extended UTAUT model. H, hypothesis. *R*^2^, *R* square.

Figure 1 illustrates a hypothetical model involving the UTAUT model, ISSM, and variable TMS to evaluate the factors that impact medical staff’s behavioral intention to use AI-CDSS.

## 2 Methods

### 2.1 Participants and data collection

Participants including nurses and clinicians were from three grade-A tertiary hospitals in Shanxi province, where the AI-CDSS for VTE prevention has been introduced or intended to be implemented. Grade-A tertiary hospitals were evaluated and classified by the local provincial health administrative department. Eligible participants must: (1) work in the hospital for at least 1 year; (2) have a registered license; (3) be volunteers. We excluded nurses and clinicians in the internship or advanced training period.

Participants were recruited through convenient sampling. The survey was conducted both online and offline from 15th October to 10th November 2023. We briefly introduced the research to participants and acquired their informed permission at the beginning. Participants completed surveys online through the survey platform Wenjuanxing^1^. We requested hospital administrators to send questionnaires to the working contact group for nurses and clinicians. Simultaneously, some participants accepted the survey by printed questionnaires.

We distributed 700 questionnaires for the study. After a manual exclusion, including incomplete, duplicate, and missing questionnaires, 510 valid questionnaires eventually remained (valid response rate = 72.86%, 510/700).

### 2.2 Research instrument

The questionnaire was to investigate what factors were influencing healthcare workers’ acceptance of AI-CDSS for VTE prevention. The questionnaire contained two sections. The first section included basic information, involving age, gender, education, occupation, length of work, and so on. The second section contained 24 items covering 7 variables based on the extended UTAUT model: top management support, information quality, system quality, social influence, and effort expectancy, performance expectancy, behavioral intention. Each item was assessed on a 5-point Likert scale varying from 1 indicating strong disagreement to 5 indicating strong agreement. The measurement items of research variables are shown in Supplementary material.

A questionnaire needed several steps to guarantee its quality. To develop the instrument, we adapted scales from previous studies (29, 34, 38, 42, 46, 47, 51–53), and modified expressions to match the context of this study. Before launching the survey, 10 professionals from top-level positions within their hospitals were interviewed to evaluate the questionnaire’s clarity, contextual relevance, logical consistency, and terminology to improve the adapted items. Based on their feedback, additional modifications and adjustments were made to improve the instrument. Then, the revised questionnaires were sent to 75 clinicians and nurses for the pre-test. We made several modifications and ensured the final questionnaire. The pre-test findings suggested Cronbach’s *α* is in the range of 0.861 to 0.983, which is ≥0.7, showing the reliability of the items (54).

In UTAUT (29, 51–53), the performance expectancy variable had four items, the effort expectancy variable had three items, the social influence variable had four items, and the behavioral intention variable contained three items. The measuring constructs in ISSM (34, 38, 42) included information quality (three items), and system quality (four items). Finally, top management support (46, 47) was measured using three items.

### 2.3 Statistical analysis

The data was analyzed using structural equation modeling. This study followed the two-stage analytical method (55). We assessed the validity and reliability of the measurement model in the first phase. We assessed reliability by the composite reliability (CR), Cronbach’s alpha, and item-loadings of each item, convergent validity (CV) by the Average Variance Extracted (AVE), and discriminant validity (DV) by the square roots of AVEs (55, 56). The second phase involved examining the relationships between the constructs and evaluating structural equation model fitting. We did a analysis on the whole healthcare workers and a multi-group analysis on clinicians and nurses, respectively. We assessed the goodness-of-fit index (GFI), the incremental fit index (IFI), and so on for the fit of the model (55, 56). We applied AMOS 21.0 software to evaluate the significance of all hypotheses by analyzing *T*-statistics, *p*-values, and path coefficients. Data were imported into AMOS21.0 for analysis. *p*-value ≤ 0.05 indicated statistical significance (two-tailed).

## 3 Results

### 3.1 Descriptive statistical analysis

In total, 510 participants were valid responses, including 96 male and 414 female. The sample comprised 186 clinicians (36.4%) and 324 nurses (63.6%). About half (46.7%, *n* = 238) were aged 26 to 35 years, followed by 36 to 45 years (26.3%, *n* = 134). Most participants (68.4%, *n* = 349) obtained an undergraduate degree, followed by a master’s degree or higher (29.2%, *n* = 149), and a junior college degree or lower (2.4%, *n* = 12). Concerning the working experience of participants in this study, 31.8% (*n* = 162) worked less than 5 years, 18% (*n* = 92) worked 5–10 years, 28.4% (*n* = 145) worked 10–15 years, 7.1% (*n* = 36) worked 15–20 years, and 14.7% (*n* = 75) worked more than 20 years. The detailed results are shown in Table 1.

**Table 1:** Profiles of respondents (*N* = 510).

Variable	Description	*N*	%
Gender	Man	96	18.8
	Woman	414	81.2
Age	25 or below	70	13.7
	26—35	238	46.7
	36—45	134	26.3
	46—55	62	12.1
	56 or above	6	1.2
Education	Junior College and below	12	2.4
	Bachelor	349	68.4
	Master or above	149	29.2
Occupation	Clinician	186	36.4
	Nurse	324	63.6
Length of working (year)	0—5	162	31.8
	5—10	92	18
	10—15	145	28.4
	15—20	36	7.1
	20 or above	75	14.7
Whether use AI-CDSS or not	Yes	232	45.5
	No	278	54.5

### 3.2 Measurement model analysis

Reliability assesses the internal consistency and stability of the questionnaire (56). CR, Cronbach’s *α*, and standardized factor loading were used to evaluate all variables’ reliability. Cronbach’s coefficients varied from 0.901 to 0.973, all of which were greater than 0.7 (56). The lowest CR value in all constructs was 0.915, which was more than 0.7 (57). The lowest standardized factor loading in all items was 0.593, greater than 0.5 (54). CV was measured by AVE and item loading in this study (57). AVEs of all constructs were greater than 0.5 (57). Therefore, reliability and CV were ensured in this model. Table 2 shows the detailed results.

**Table 2:** Reliability and convergent validity results.

Construct	Items	Item loading	Cronbach’s α	Composite reliability (CR)	Average variance extracted (AVE)
PE^a^	PE1	0.886	0.956	0.957	0.848
	PE2	0.938			
	PE3	0.937			
	PE4	0.921			
EE^b^	EE1	0.955	0.947	0.950	0.863
	EE2	0.955			
	EE3	0.875			
SI^c^	SI1	0.593	0.901	0.915	0.735
	SI2	0.895			
	SI3	0.955			
	SI4	0.935			
SQ^d^	SQ1	0.875	0.956	0.957	0.847
	SQ2	0.908			
	SQ3	0.954			
	SQ4	0.943			
IQ^e^	IQ1	0.943	0.973	0.974	0.926
	IQ2	0.976			
	IQ3	0.967			
TMS^f^	TMS1	0.905	0.952	0.952	0.870
	TMS2	0.955			
	TMS3	0.937			
BI^g^	BI1	0.922	0.959	0.960	0.888
	BI2	0.953			
	BI3	0.952			

^aPE, performance expectancy.^

^bEE, effort expectancy.^

^cSI, social influence.^

^dSQ, system quality.^

^eIQ, information quality.^

^fTMS, top management support.^

^gBI, behavioral intention.^

The square roots of AVE could evaluate DV (56). The results showed that all the correlation coefficients of different variables were lower than the square roots of AVEs. Thus, DV is ensured. The statistics results are shown in Table 3.

**Table 3:** Discriminant validity results.

Constructs	PE^b^	EE^c^	SI^d^	SQ^e^	IQ^f^	TMS^g^	BI^h^
Performance expectancy	*0.921* ^a^						
Effort expectancy	0.595	*0.929*					
Social influence	0.764	0.507	*0.857*				
System quality	0.500	0.429	0.417	*0.920*			
Information quality	0.520	0.432	0.428	0.738	*0.962*		
Top management support	0.685	0.593	0.557	0.519	0.579	*0.933*	
Behavioral intention	0.794	0.677	0.6	0.595	0.597	0.767	*0.942*

^aData using italics on the leading diagonals is the square root of AVE. The other data are the correlation coefficients among the variables.^

^bPE, performance expectancy.^

^cEE, effort expectancy.^

^dSI, social influence.^

^eSQ, system quality.^

^fIQ, information quality.^

^gTMS, top management support.^

^hBI, behavioral intention.^

### 3.3 Structural model analysis

**Figure 2:**
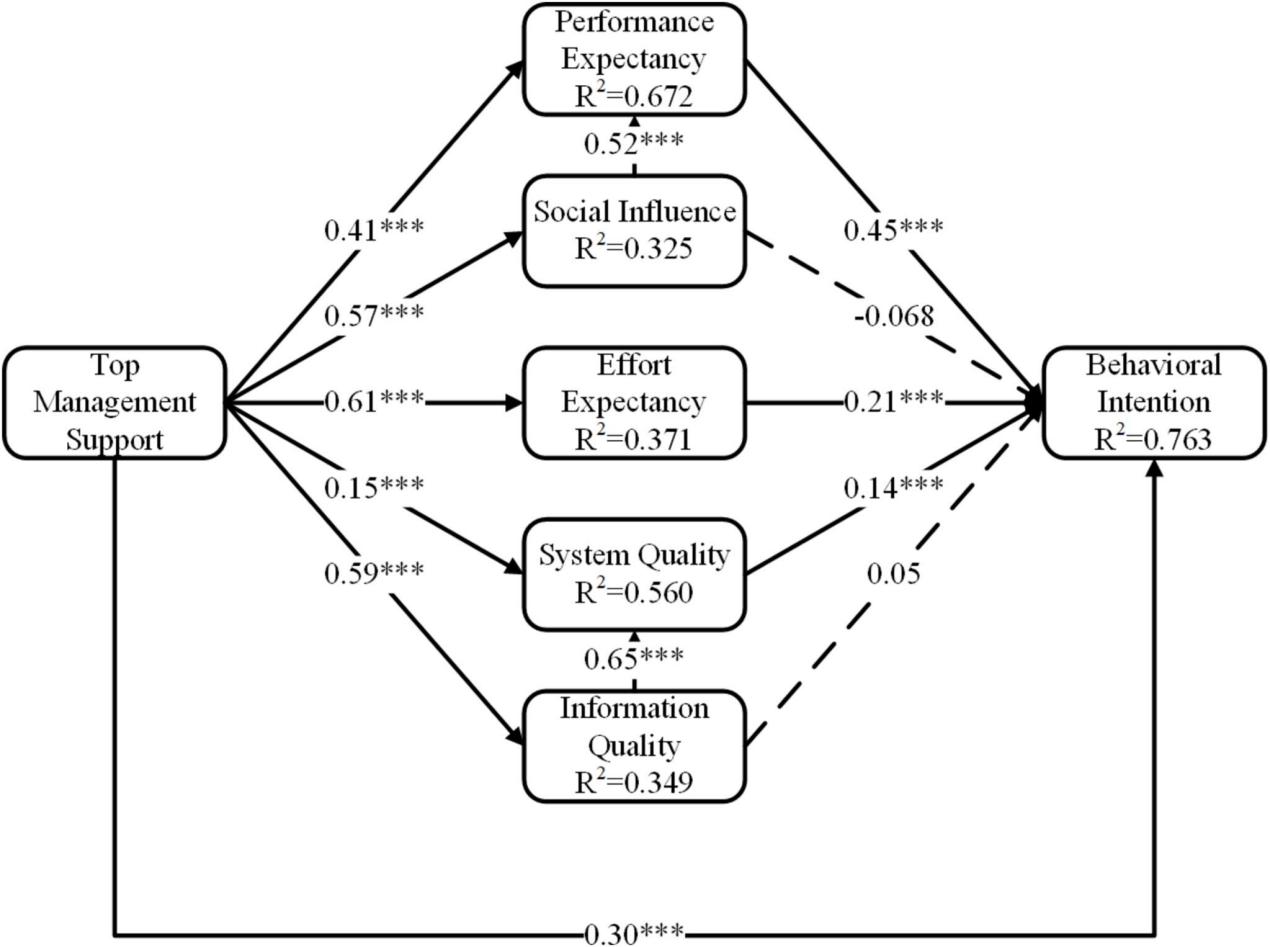
The final extended UTAUT model. ***p < 0.001.

We first analyzed the whole healthcare workers’ intention and its influencing factors based on the structural equation modeling. Figure 2 and Table 4 indicate hypothesis measuring results of whole healthcare workers, the model’s fit statistics revealed a good model fit (*χ*^2^/df = 2.599, RMSEA = 0.056, IFI = 0.976, GFI = 0.910, CFI = 0.976, and TLI = 0.972).

**Table 4:** Estimation results for hypothesis (model 1).

Hypothesis	Path	Standardized coefficient	*T*-value	*P*-value	Test results
H1	Performance expectancy → Behavioral intention	0.445	9.246	<0.001	Supported
H2	Effort expectancy → Behavioral intention	0.207	6.467	<0.001	Supported
H3	Social influence → Behavioral intention	−0.068	−1.733	0.09	Non-supported
H4	Social influence → Performance expectancy	0.517	10.486	<0.001	Supported
H5	System quality → Behavioral intention	0.135	3.508	<0.001	Supported
H6	Information quality → Behavioral intention	0.050	1.254	0.21	Non-supported
H7	Information quality → System quality	0.650	14.953	<0.001	Supported
H8	Top management support → Behavioral intention	0.295	6.272	<0.001	Supported
H9	Top management support → Performance expectancy	0.406	10.867	<0.001	Supported
H10	Top management support → Effort expectancy	0.609	15.571	<0.001	Supported
H11	Top management support → Social influence	0.570	10.803	<0.001	Supported
H12	Top management support → System quality	0.150	3.722	<0.001	Supported
H13	Top management support → Information quality	0.591	14.961	<0.001	Supported

Specifically, performance expectancy (*β* = 0.45, *P* <0.001), effort expectancy (*β* = 0.21, *P* < 0.001), system quality (*β* = 0.14, *P* < 0.001), and top management support (*β* = 0.30, *P* < 0.001) positively influenced behavioral intention. Additionally, Social influence positively influenced performance expectancy (*β* = 0.52, *P* < 0.001), and information quality positively influenced system quality (*β* = 0.65, *P* < 0.001). Third, top management support was the crucial antecedent of performance expectancy (*β* = 0.41, *P* < 0.001), social influence (*β* = 0.57, *P* < 0.001), effort expectancy (*β* = 0.61, *P* < 0.001), system quality (*β* = 0.15, *P* < 0.001) and information quality (*β* = 0.59, *P* < 0.001).

Then, we further did the multi-group analysis of clinicians’ and nurses’ intentions. However, the results showed that the differences were significant among different occupations [*P* (structural weights) = 0.00 < 0.05, *P* (Structural residuals) = 0.00 < 0.05, *P* (measurement residuals) = 0.00 < 0.05]. Therefore, there were occupation differences in the model. Table 5 shows the multi-group analysis results of clinicians, Table 6 shows the multi-group analysis results of nurses.

**Table 5:** Estimation results for hypothesis among clinicians (model 2).

Hypothesis	Path	Standardized coefficient	*T*-value	*P*-value	Test results
H1	Performance expectancy → Behavioral intention	0.493	6.432	<0.001	Supported
H2	Effort expectancy → Behavioral intention	0.276	5.126	<0.001	Supported
H3	Social influence → Behavioral intention	−0.189	−3.303	<0.001	Non-supported
H4	Social influence → Performance expectancy	0.459	6.879	<0.001	Supported
H5	System quality → Behavioral intention	0.092	1.321	0.187	Non-supported
H6	Information quality → Behavioral intention	0.146	2.244	0.025	Supported
H7	Information quality → System quality	0.625	9.151	<0.001	Supported
H8	Top management support → Behavioral intention	0.224	2.944	0.003	Supported
H9	Top management support → Performance expectancy	0.490	8.258	<0.001	Supported
H10	Top management support → Effort expectancy	0.624	9.466	<0.001	Supported
H11	Top management support → Social influence	0.443	5.736	<0.001	Supported
H12	Top management support → System quality	0.264	4.361	<0.001	Supported
H13	Top management support → Information quality	0.535	7.861	<0.001	Supported

**Table 6:** Estimation results for hypothesis among nurses (model 3).

Hypothesis	Path	Standardized coefficient	*T*-value	*P*-value	Test results
H1	Performance expectancy → Behavioral intention	0.368	5.638	<0.001	Supported
H2	Effort expectancy → Behavioral intention	0.162	4.123	<0.001	Supported
H3	Social influence → Behavioral intention	0.055	0.884	0.376	Non-supported
H4	Social influence → Performance expectancy	0.639	8.239	<0.001	Supported
H5	System quality → Behavioral intention	0.161	3.463	<0.001	Supported
H6	Information quality → Behavioral intention	−0.021	−0.414	0.679	Non-supported
H7	Information quality → System quality	0.669	11.925	<0.001	Supported
H8	Top management support → Behavioral intention	0.335	5.510	0.003	Supported
H9	Top management support → Performance expectancy	0.279	5.831	<0.001	Supported
H10	Top management support → Effort expectancy	0.596	11.773	<0.001	Supported
H11	Top management support → Social influence	0.664	8.436	<0.001	Supported
H12	Top management support → System quality	0.089	1.680	0.093	Non-supported
H13	Top management support → Information quality	0.622	12.444	<0.001	Supported

According to Tables 5, 6, H1, H2, H4, H7, H8, H9, H10, H11, and H13 were also supported in multi-group models. However, H3, H5, H6, and H12 were different in multi-group models. In H3, social influence did not influence nurses’ behavioral intention (*β* = 0.06, *P* = 0.376), but negatively influenced clinicians’ behavioral intention (*β* = −0.19, *p* < 0.001). In H5, system quality positively influenced nurses’ behavioral intention (*β* = 0.16, *p* < 0.001), but did not affect clinicians’ behavioral intention in the clinicians’ model (*β* = 0.09, *P* = 0.187). In H6, information quality did not influence nurses’ behavioral intention (*β* = −0.02 , *p* = 0.679), but positively influenced clinicians’ behavioral intention (*β* = 0.15, *p* = 0.025). In H12, top management support influenced system quality in the clinicians’ model (*β* = 0.26, *P* < 0.001), but the relation was not obvious in the nurses’ model (*β* = 0.09, *p* = 0.093).

## 4 Discussion

AI-CDSS in this study is based on natural language processing, machine learning, knowledge mapping, and other technologies, which can keep continuous learning, provide real-time and dynamic decision support and so on. For clinicians, the system could provide them with electronic preventive reminders on preventive suggestions (19). Specifically, based on natural language processing technology, AI-CDSS could automatically and dynamically extract, identify and evaluate patients’ information on VTE risk, and bleeding risk in hand-typed medical records. When the assessment results showed the patient was at increased risk, AI-CDSS would give clinicians corresponding preventive pop-up reminders based on the ACCP guidelines (9th edition) and expert consensus. For nurses, the system could also help them conduct a comprehensive VTE risk assessment for patients after a preliminary judgment from clinicians, which strengthens the communication between doctors and nurses about VTE risk assessment. In addition, the system prompts nurses to implement clinicians’ instructions. Third, nurses could use the patient education materials generated by the system to educate patients and their families about VTE. For hospital administrators, the system could conduct real-time statistics on the prevention and control of VTE in the whole hospital, including key indicators such as VTE risk assessment rate, bleeding risk assessment rate and implementation rate of mechanical prevention and drug prevention, which could be helpful for them to know about the implementation in the whole hospital and then to manage. A brief introduction of key takeaways for the use of AI-CDSS is in Supplementary Table S1.

This study combined UTAUT, ISSM, and the top management support variable to explore factors influencing medical staff’s acceptance of AI-CDSS for VTE prevention. Firstly, top management support, effort expectancy, and performance expectancy, all positively impacted the medical staff’s behavioral intention. Secondly, top management support influenced information quality, social influence, effort expectancy, and performance expectancy. Thirdly, information quality positively affected clinicians’ behavioral intention, system quality positively influenced nurses’ behavioral intention. Fourth, social influence had no significant effect on the intention of nurses to use the system and even had a negative effect on behavioral intention of clinicians.

Statistical results showed performance expectancy and effort expectancy, two core constructs in UTAUT, positively impacted behavioral intention, which supports previous research (25, 32), and the results also showed that top management support positively influenced behavioral intention. Performance expectancy refers to the usefulness of AI-CDSS that healthcare workers think. For example, when healthcare workers assess the risk of VTE, AI-CDSS can automatically assess and remind them in time (58, 59), replacing the previous manual assessment process. Therefore, healthcare workers may consider AI-CDSS as an effective and efficient solution, which provides great convenience for them to manage patients with or at risk of VTE, and they may be more willing to use it. Effort expectancy positively influenced behavioral intention. Effort expectation refers to the degree of ease in learning AI-CDSS that healthcare workers perceive. For example, when healthcare workers perceive AI-CDSS as easy to learn, they may prefer to use it. In fact, AI-CDSS has a simple and intuitive interface. It is easy to learn and operate, and this learning and use process does not take medical staff too much time and energy. Top management support refers to the human, material and financial support provided by the hospital leadership to promote the introduction and use of AI-CDSS. For example, when hospital leadership supports to introduce AI-CDSS, takes measures such as rewards and punishments or propaganda and education, medical staff will be more likely to use AI-CDSS.

Statistical results also showed top management support positively influenced effort expectancy and performance expectancy, social influence, and information quality, which is similar to earlier research (40). For example, when hospital leadership take advertisement or education measures, medical staff will be encouraged to understand the practical function of AI-CDSS for VTE prevention and control, thus recognizing the usefulness of AI-CDSS, and performance expectancy will be enhanced. Similarly, regular advertisement and education measures taken by hospital leadership are beneficial for medical staff to understand the operation and use of AI-CDSS and improve their familiarity with AI-CDSS. Thus, the medical staff’s perceived ease of use will be enhanced. Social influence in our research refers to the influence of members of the VTE prevention and control group or some colleagues in the hospital on medical staff intention to use AI-CDSS. For example, when hospital leadership publish policies for the implementation of AI-CDSS, some medical staff such as members of the VTE group may be influenced by the policies published and then suggest other healthcare workers use AI-CDSS. Information quality in this study refers to whether AI-CDSS provides real-time, accurate, and comprehensive information. If top management could introduce high-quality AI-CDSS for VTE prevention and control, therefore the quality of information would be ensured.

Multi-group analysis showed a difference between clinicians and nurses. For clinicians, information quality significantly affected behavioral intention to use the new system, while the impact of system quality was not significant. Conversely, for nurses, system quality significantly influenced behavioral intention, and the effect of information quality was not apparent. This difference may be due to the different responsibilities of clinicians and nurses when they work (60). Clinicians are primarily responsible for clinical decision-making and therefore focus more on whether the system can accurately and comprehensively capture patient information on VTE; nurses mainly carry out medical orders and thus pay more attention to the response speed and reliability of the system.

The multi-group analysis also found social influence insignificantly influenced intention among nurses. This finding is different from most previous studies (38, 61). Only several studies had similar results to our study (33, 62). One possible explanation is that the regulatory issues are of importance to the medical staff’s acceptance of AI-CDSS (63), but the regulatory issues provided by VTE groups in clinical departments or hospital management departments are inadequate and lacking during the implementation of AI-CDSS in the hospitals surveyed this time. Therefore, social influence will not influence the work of medical staff directly. Another possible explanation is due to the great work pressure healthcare workers face (64, 65). It means that even if colleagues (such as members of the VTE group in the hospital) recommend the use of the new system, the intentions of healthcare workers were less impacted. However, the multi-group analysis found that social influence negatively influenced behavioral intention among clinicians. On the one hand, clinicians are under great professional pressure, on the other hand, the profession requires them to maintain a high sense of autonomy and responsibility in clinical work (66). Therefore, if clinicians comprehensively judge that the new system cannot meet the clinical needs, the recommendation from VTE team members may even be counterproductive.

There are some other findings. We found that top management support positively influenced system quality in the clinicians’ model, but the relation was not obvious in the nurses’ model. This may be due to that with the increase in the number of paths in the model, the complexity of the model will also increase, which may lead to a decrease in the significance of some paths in the nurses’ model. We also found that social influence positively influenced performance expectancy, which means that the suggestions by colleagues around healthcare workers to use AI-CDSS will make them feel the usefulness of AI-CDSS; information quality positively influenced system quality among healthcare workers, which means that the function of capturing information accurately and completely affects the quality of the system.

In addition, comparing our study on AI-CDSS to the studies on some technologies with a low degree of artificial intelligence, there is an interesting finding. In our research on AI-CDSS, the coefficient values of performance expectation and effort expectation to intention are larger in the model. However, in the studies on some technologies with a low degree of artificial intelligence, such as the studies of intention to use the Emergency Department (ED) wait-times website (67) and mobile payments (68), the influence of performance expectation and effort expectation to behavioral intention in the model is low or even is not significant in the model. This shows that the perceived usefulness and ease of use of technologies with a high degree of artificial intelligence, meaning with the ability to reason and problem-solve, have a greater impact on people’s behavioral intention, highlighting the importance and influence of high intelligence of AI.

This research has limitations. To begin, the study is a cross-sectional study, which investigated the correlation among variables but not a causal relationship. Future research could consider longitudinal designs to understand the causal relationships between variables better. In addition, participants were only recruited from three hospitals in Shanxi, China. The regional limitation of the sample restricts the generalizability of the research results. Therefore, it is suggested that future research investigate the effectiveness of AI-CDSS in different regions and diverse levels of hospitals to improve its predictive power. Third, this study employed a convenience sampling method, which may lead to selection bias to some extent. The proportion of female participants in this study was relatively high, affecting the generalizability of the results. Therefore, in future research, it is suggested to increase the sample size, adopt multistage sampling methods, or stratified sampling methods to reduce bias and enhance the representativeness of the sample.

## 5 Conclusion

Our study aimed to investigate what factors were influencing healthcare workers’ acceptance of AI-CDSS for VTE prevention based on empirically extended UTAUT with the addition of SQ and IQ variables in ISSM and the TMS variable. The results revealed that TMS, EE, and PE positively influenced medical staff’s behavioral intention. We also found that TMS is the antecedent of PE, EE, SI, and IQ. In addition, we found that IQ positively influenced clinicians’ behavioral intention, SQ positively influenced nurses’ behavioral intention; SI did not influence nurses’ behavioral intention, but SI negatively influenced clinicians’ behavioral intention. This study constructs a model that is more suitable for exploring the influencing factors of medical staff’s intention to use AI-CDSS for VTE prevention and control and expands the UTAUT model, which is a supplement and enrichment to previous related studies. In addition, this study offers a reference for hospital managers when evaluating future developments and facilitates the implementation of AI-CDSS for VTE prevention. Specifically, when hospitals tend to introduce new systems such as AI-CDSS, it is suggested to consider system quality including response speed, interface design, integration and compatibility of the system, consider the information quality of the system such as if the system could provide up-to-date, accurate, and comprehensive information, consider the supports by hospital leadership such as funds and resources support or material and spiritual incentives, which helps improve the perceived usefulness and ease of healthcare workers, thus promoting the implementation of the new system in hospitals.

## Data Availability

The original contributions presented in the study are included in the article/Supplementary material, further inquiries can be directed to the corresponding author.

## Footnotes

^1^ https://www.wjx.cn/
