# Supplementary material for: Acceptance of artificial intelligence clinical assistant decision support system to prevent and control venous thromboembolism among healthcare workers: an extend Unified Theory of Acceptance and Use of Technology Model
Source: Front Med (Lausanne). 2025 Feb 11;12:1475577. doi: 10.3389/fmed.2025.1475577 (PMC11850527; doi:10.3389/fmed.2025.1475577)
Supplement: Supplementary file 1 [file Table_1.docx]

Supplementary Material

| Medical staff | The use of AI-CDSS |
| --- | --- |
| Clinicians | Help conduct VTE risk assessment automatically. |
|  | Provide electronic preventive reminders on personalized preventive suggestions based on guidelines and expert consensus. |
| Nurses | Help conduct a comprehensive VTE risk assessment after a preliminary judgment from clinicians. |
|  | Prompt to implement clinicians' instruction in-time. |
|  | Offer the patient personalized education materials about VTE. |
| Hospital administrators | Conduct real-time statistics on the prevention and control of VTE in the whole hospital to facilitate management and improvement. |

Supplementary Table S1: key takeaways for the implementation of AI-CDSS
