# Supplementary material for: Acceptance of artificial intelligence clinical assistant decision support system to prevent and control venous thromboembolism among healthcare workers: an extend Unified Theory of Acceptance and Use of Technology Model
Source: Front Med (Lausanne). 2025 Feb 11;12:1475577. doi: 10.3389/fmed.2025.1475577 (PMC11850527; doi:10.3389/fmed.2025.1475577)
Supplement: Supplementary file 2 [file Data_Sheet_1.docx]

Supplementary Material

Measurement items of research variables

| variable | Serial number | Measurement items |
| --- | --- | --- |
| social influence | SC1 | The support of the hospital (policy decision, publicity and promotion) will affect my use of AI-CDSS for VTE prevention. |
|  | SC2 | I am willing to use AI-CDSS when people around me recommend me to use it. |
|  | SC3 | When colleagues around me (members of the VTE group in the hospital) think that I should use the AI-CDSS for VTE prevention, I am willing to use it. |
|  | SC4 | When my colleagues (doctors, and nurses in the hospital) think I should use AI-CDSS for VTE prevention, I am willing to use it. |
| Performance expectation | PE1 | Using AI-CDSS helps me to dynamically evaluate and monitor the risk of VTE in patients. |
|  | PE2 | Using AI-CDSS helps me to make clinical decisions to prevent VTE (taking different measures according to risk stratification). |
|  | PE3 | Using AI-CDSS can improve the quality of my work. (VTE evaluation and clinical decision-making work) |
|  | PE4 | Using AI-CDSS helps me improve my work efficiency. (VTE evaluation and clinical decision-making work) |
| Effort  Expectancy | EE1 | I think it is easy to learn the operation of AI-CDSS. |
|  | EE2 | I think it is easy to skillfully use AI-CDSS for VTE prevention. |
|  | EE3 | Using AI-CDSS when working won't take up too much energy. |
| Behavioral  Intention | BI1 | I am willing to use AI-CDSS for VTE prevention in the future. |
|  | BI2 | If the AI-CDSS is introduced, I will use it as much as possible. |
|  | BI3 | I would like to recommend others to use AI-CDSS. |
| System quality | SQ1 | The response speed of AI-CDSS affects my use intention. |
|  | SQ2 | Whether the interface design of AI-CDSS is clear and reasonable affects my use intention. |
|  | SQ3 | The reliability of AI-CDSS (such as data saving and security) affects my use intention. |
|  | SQ4 | The integration and compatibility of AI-CDSS affect my use intention. |
| Information quality | IQ1 | Whether AI-CDSS provides real-time and up-to-date information affects my use intention. |
|  | IQ2 | Whether AI-CDSS provides accurate information affects my use intention. |
|  | IQ3 | Whether the AI-CDSS provides sufficient and comprehensive information affects my use intention. |
| Top management support | TMS1 | I believe the hospital top management know the benefits of introducing AI-CDSS. |
|  | TMS2 | Hospital top management will support and encourage the implementation of AI-CDSS. |
|  | TMS3 | Hospital top management will ensure funds and resources sufficient to facilitate the implementation and operation of AI-CDSS. |
